# Engineered Nanovesicles from Fibroblasts Modulate Dermal Papillae Cells In Vitro and Promote Human Hair Follicle Growth Ex Vivo

**DOI:** 10.3390/cells11244066

**Published:** 2022-12-15

**Authors:** Ramya Lakshmi Rajendran, Prakash Gangadaran, Mi Hee Kwack, Ji Min Oh, Chae Moon Hong, Madhan Jeyaraman, Young Kwan Sung, Jaetae Lee, Byeong-Cheol Ahn

**Affiliations:** 1 Department of Nuclear Medicine, School of Medicine, Kyungpook National University, Daegu 41944, Republic of Korea; 2 BK21 FOUR KNU Convergence Educational Program of Biomedical Sciences for Creative Future Talents, Department of Biomedical Sciences, School of Medicine, Kyungpook National University, Daegu 41944, Republic of Korea; 3 Department of Immunology, School of Medicine, Kyungpook National University, Daegu 41944, Republic of Korea; 4 Department of Nuclear Medicine, Kyungpook National University Hospital, Daegu 41944, Republic of Korea; 5 Department of Orthopaedics, ACS Medical College and Hospital, Dr MGR Educational and Research Institute University, Chennai 600056, Tamil Nadu, India; 6 Department of Biotechnology, School of Engineering and Technology, Sharda University, Greater Noida 201310, Uttar Pradesh, India

**Keywords:** alopecia, dermal papilla, extracellular vesicles, engineered nanovesicles

## Abstract

Alopecia is a common medical condition affecting both sexes. Dermal papilla (DP) cells are the primary source of hair regeneration in alopecia patients. Therapeutic applications of extracellular vesicles (EVs) are restricted by low yields, high costs, and their time-consuming collection process. Thus, engineered nanovesicles (eNVs) have emerged as suitable therapeutic biomaterials in translational medicine. We isolated eNVs by the serial extrusion of fibroblasts (FBs) using polycarbonate membrane filters and serial and ultracentrifugation. We studied the internalization, proliferation, and migration of human DP cells in the presence and absence of FB-eNVs. The therapeutic potential of FB-eNVs was studied on ex vivo organ cultures of human hair follicles (HFs) from three human participants. FB-eNVs (2.5, 5, 7.5, and 10 µg/mL) significantly enhanced DP cell proliferation, with the maximum effect observed at 7.5 µg/mL. FB-eNVs (5 and 10 µg/mL) significantly enhanced the migration of DP cells at 36 h. Western blotting results suggested that FB-eNVs contain vascular endothelial growth factor (VEGF)-a. FB-eNV treatment increased the levels of PCNA, pAKT, pERK, and VEGF-receptor-2 (VEGFR2) in DP cells. Moreover, FB-eNVs increased the human HF shaft size in a short duration ex vivo. Altogether, FB-eNVs are promising therapeutic candidates for alopecia.

## 1. Introduction

Extracellular vesicles (EVs) are membrane-enclosed nanoscale particles and ubiquitous nanosized vesicles that are released from most cells into the extracellular space. They include small EVs, exosomes, microvesicles, and apoptotic bodies. Exosomes (40–200 nm) are released from the intracellular membranous compartment, whereas microvesicles (40–1000 nm) and apoptotic bodies (50–5000 nm) are released by the blebbing of the cellular membrane and apoptotic cells, respectively [1,2]. EVs are secreted by cells to exchange biological information, such as lipids, proteins, and nucleic acids, between local and distant cells and contribute to a diverse array of physiological and pathological developments [3]. Because EVs can impact the behavior of recipient cells, they serve as a potential source of therapeutics.

Several therapeutic strategies, such as gene therapy, cell engineering, cell therapy, tissue engineering scaffolds, and cell-based therapy, are vital for tissue repair in regenerative medicine [4]. Recently, cell-free therapy has emerged as a topic of interest among research and clinical communities. The regenerative properties of EV-based therapies are similar to or superior to those of cell therapy [5,6]. EV-based therapies are gaining increasing attention in the field of regenerative medicine, and EVs could be a good alternative to conventional therapeutic biomaterials in translational medicine.

Hair loss (alopecia) is a common medical disorder affecting both sexes. Numerous studies conducted to assess the therapeutic applications of EVs obtained from different cells (dermal papilla (DP), stem cells, macrophages, and fibroblasts) in treating hair loss have demonstrated that EV-based treatments can facilitate hair growth [7]. Studies have frequently used naturally released EVs to treat several diseases, including hair loss. However, preclinical and clinical research on the use of EVs is significantly limited owing to the low production of EVs by cells [8,9].

Researchers have developed engineered nanovesicles (eNVs) from cells to overcome this constraint, and their mass production has led to their increased clinical application [10,11]. Only two studies have demonstrated that eNVs synthesized from macrophages and neural progenitor cells can induce hair growth [12,13]; however, no studies have reported the production of eNVs from fibroblasts. Compared to stem cells (from bone marrow or adipose), macrophages, or neural progenitor cells, fibroblast cells are associated with skin and hair growth [7]. In addition, fibroblast cells have been demonstrated to respond to injury and hair cycling in the skin [14]. Therefore, we identified the effects of engineered fibroblast-NVs (FB-eNVs) on human DP cells and hair follicles (HFs).

## 2. Materials and Methods

### 2.1. Cell Culture

The mouse fibroblast cell line L929 (L-929, derivative of strain L) was kindly gifted by Dr. Yong Hyun Jeon (Daegu-Gyeongbuk Medical Innovation Foundation, Daegu, Republic of Korea). Cells were cultured in Dulbecco’s modified Eagle medium (DMEM), high-glucose (HyClone, Logan, UT, USA), and supplemented with 10% fetal bovine serum (FBS; HyClone) and 1% penicillin–streptomycin (Gibco, Carlsbad, CA, USA) at 37 °C with 5% CO_2_.

### 2.2. Primary Culture of DP Cells

The HFs of the anagen phase were collected from the scalp skin as soon as informed consent was obtained from the corresponding patients. This study was approved by the Medical Ethics Committee of the Kyungpook National University and Hospital (Daegu, Republic of Korea) and was performed in accordance with the guidelines and principles of the Declaration of Helsinki. DP cells were isolated from the dissected HFs of the bulbs and cultured in DMEM, low-glucose (HyClone), supplemented with penicillin (100 U/mL), streptomycin (100 μg/mL), bovine fibroblast growth factor, and 20% heat-inactivated FBS at 37 °C. Explants were isolated for 7 days, the medium was changed every 3 days, and the collected DP cells were plated in culture dishes (100 mm) in DMEM low-glucose supplemented with heat-inactivated FBS. The sub-cultures were performed according to the percentage of the confluence of cells, and passage number 2 was applied.

### 2.3. Generation of Engineered Nanovesicles from Fibroblasts

L929 cells were extruded five times through 10-μm, 5-μm, and 1-μm polycarbonate membrane filters (Nuclepore, Whatman, Inc., Clifton, NJ, USA) using a mini-extruder (Avanti Polar Lipids, Birmingham, AL, USA). The isolated samples were filtered through a 0.45-μm syringe filter and ultracentrifuged at 100,000× *g* for 1 h (Beckman Coulter, Brea, CA, USA). Subsequently, two-step iodixanol (OptiPrep, Sigma, Oliver Township, MI, USA) density gradient ultracentrifugation was performed at 120,000× *g* for 3 h. The sample was collected from the crossing point of the 60% and 20% layers of iodixanol and stored at −80 °C.

### 2.4. Western Blot Analysis

To perform Western blotting, lysates of fibroblasts/DP cells/nanovesicles were prepared in radioimmunoprecipitation assay (RIPA) buffer (Thermo Fisher Scientific, Waltham, MA, USA). Samples were loaded equally and separated by 10% sodium dodecyl sulfate–polyacrylamide gel electrophoresis. The samples were subsequently transferred to polyvinylidene difluoride (PVDF) membranes (Millipore, Burlington, MA, USA). The blots were probed with primary antibodies (Abcam: Alix, GM130, and Calnexin; Cell Signaling Technology: HSP90, Flotillin-1, PCNA, VEGFR-2, pAKT, AKT, pERK ERK, and β-actin), followed by incubation with the secondary antibody conjugated with horseradish peroxidase (Cell Signaling Technology, Danvers, MA, USA). The signals were observed using enhanced chemiluminescence (GE Healthcare, Chicago, IL, USA) according to the manufacturer’s instructions.

### 2.5. Transmission Electron Microscopy (TEM)

The morphology of FB-eNVs was observed using TEM. FB-eNVs were mixed with 100 µL of 2% paraformaldehyde. Next, 5 µL of FB-eNVs pellets were attached to the Formvar carbon-coated grids and covered with a protective material such as aluminum foil for 20 min to avoid any damage/dryness to the sample. Approximately 100 µL of phosphate-buffered saline (PBS) was added to a sheet of parafilm, and grids were transferred onto drops of PBS using sterile forceps for washing. Subsequently, the samples were transferred to 50 µL of 1% glutaraldehyde, incubated at 25 to 30 °C for 5 min, and subsequently washed with distilled water for 2 min. FB-eNVs were stained with 2% uranyl acetate. These steps were repeated seven more times, and the samples were allowed to completely dry before observation under an HT 7700 transmission electron microscope (Hitachi, Tokyo, Japan) to view the size of FB-eNVs.

### 2.6. FB-eNV Uptake Assay

To examine the internalization of FB-eNVs, 10 μg of FB-eNVs was incubated with Dil Stain (1,1′-Dioctadecyl-3,3,3′,3′-Tetramethylindocarbocyanine perchlorate [‘DiI’; DiIC18(3)]) for 20 min and mixed with PBS. Two-step OptiPrep density gradient ultracentrifugation was performed at 120,000× *g* for 3 h. The Dil-labeled FB-eNVs (FB-eNVs/Dil) were collected and used. DP cells (5000) were cultured on eight-well slides and incubated overnight. The next day, FB-eNVs or FB-eNVs/Dil were added to DP cells for 2 h and maintained at 37 °C in 5% CO_2_. The slides were subsequently fixed in methanol and mounted using Vectashield antifade mounting medium (Vector Laboratories, Burlingame, CA, USA), and the internalization of FB-eNVs/Dil was observed under a laser scanning microscope (LSM 800 with Airyscan, Zeiss, Oberkochen, Baden-Württemberg, Germany).

### 2.7. Cell Proliferation Assay

Cell proliferation assays were performed using the Cell Counting Kit-8 (CCK-8; Dojindo, Kumamoto, Japan). DP cells (5000 cells/well) were seeded in a 96-well plate, treated with different concentrations of FB-eNVs, and maintained at 37 °C in 5% CO_2_. After 24 h, the CCK-8 reagent (10 μL) was added to each well and samples were incubated for 2 h at 37 °C in 5% CO_2_. The optical density (OD) values were measured at 450 nm using a microplate reader.

### 2.8. In Vitro Cell Migration Assay

The DP cells (5 × 10^4^) were seeded in 6-well plates and incubated overnight at 37 °C in 5% CO_2_. The next day, a scratch wound was created using a 10-µL tip, and the detached cells were removed by replacing the media. Cells were immediately treated with FB-eNVs (5 and 10 µg/mL). Images were acquired at 0 and 36 h using the AxioVision software version 4.5 (Zeiss). Open wound distance was estimated with at least five measurements in each microscopic field of view using ZEN Lite 2.3 (Carl Zeiss, Oberkochen, Baden-Württemberg, Germany).

### 2.9. RNA Isolation and Quantitative Real-Time Polymerase Chain Reaction (qRT-PCR)

DP cells were collected after 24 h of treatment with FB-eNVs (5 and 10 µg/mL) and the RNA was isolated using the TRIzol reagent. The cDNAs were performed using a high-capacity cDNA kit (Applied Biosystems, Wakefield, MA, USA). The mRNA expression of genes was detected using qRT-PCR analysis and performed with SsoAdvanced Universal SYBR Green Supermix reagent (Bio-Rad Laboratories, Irvine, CA, USA) on an ABI-7500 detection system (Applied Biosystems, Wakefield, MA, USA) according to the manufacturer’s instructions. The sense and antisense primers are listed in Supplementary Table S1. The relative expression of mRNA was calculated using the 2^−ΔΔct^ method [15].

### 2.10. Human Hair Shaft Elongation

Human HFs were isolated and cultured as per previously described methods [16]. Briefly, surgical/biopsy samples were collected from the occipital scalps of patients (male) with androgenic alopecia during a hair transplantation procedure, with written informed consent from the patients. The Medical Ethics Committee of the Kyungpook National University Hospital (Daegu, Korea) approved all the described studies. The HFs were isolated from non-balding scalps, and the subcutaneous fat portions of the scalp skin along with the lower HFs were separated from the epidermis and dermis. Next, HFs were isolated with the help of forceps under a binocular microscope. The HFs were cultured in Williams E media without phenol red (Sigma, Oliver Township, MI, USA) at 37 °C in a humidified atmosphere with 95% O_2_ and 5% CO_2_. The HFs from three individuals were treated with FB-eNVs (0, 0.1, and 0.5 µg/mL). Hair shaft elongation was subsequently measured on days 3 and 6 using the iSolution Lite software (*i*-solution, Los Angeles, NY, USA). Macroscopic images were obtained on day 6.

### 2.11. Statistical Analysis

All data are expressed as mean ± standard deviation (SD). Differences between groups were statistically analyzed using Student’s *t*-test using Microsoft Office (Microsoft, Los Angeles, CA, USA) or GraphPad Prism version 9.4.1.681 (GraphPad Software Inc., La Jolla, CA, USA). A *p*-value < 0.05 was considered statistically significant.

## 3. Results

### 3.1. Characterization of Engineered Nanovesicles of Fibroblasts

**Figure 1:**
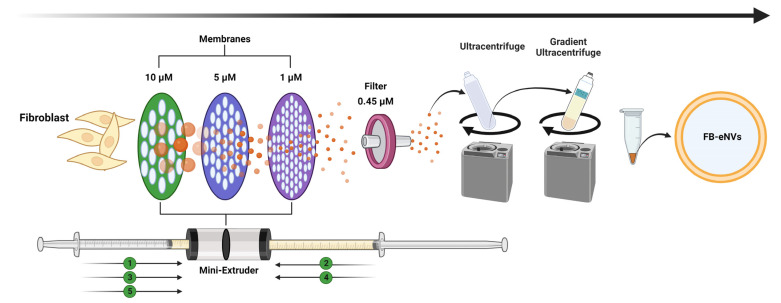
Establishment of engineered nanovesicles of fibroblasts (FB-eNVs). Schematic illustration of the generation and purification of FB-eNVs. The fibroblasts were extruded through 10-, 5-, and 1-µM membranes and subsequently filtered through 0.45-µM filters. The filtered, unpurified samples were ultracentrifuged and purified using OptiPrep density gradient ultracentrifugation. The figure was created using BioRender.com.

**Figure 2:**
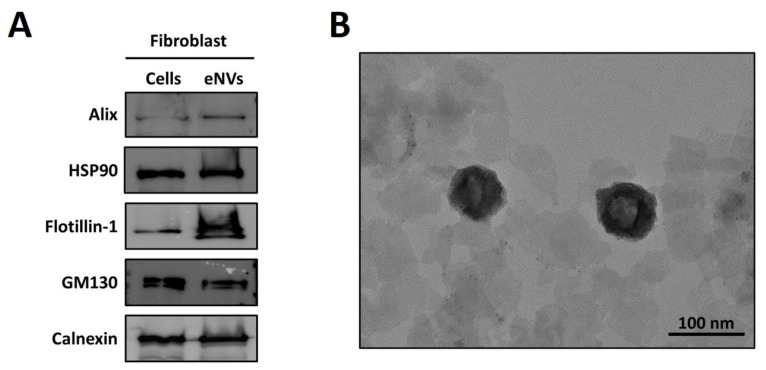
Characterization of engineered nanovesicles of fibroblasts. (**A**) Western blot analysis of fibroblasts and FB-eNV lysates for the detection of EV biomarkers (Alix, HSP90, and Flotillin-1) and cellular markers (GM130 and calnexin). (**B**) Transmission electron microscopy image of eNVs (scale bar: 100 nm).

Fibroblasts were extruded through membranes (10, 5, and 1 µM) five times, as depicted in Figure 1, and further filtered, enriched, and purified to produce FB-eNVs. EV biomarkers such as Alix (cytoplasmic proteins, regulators of vesicular trafficking process), HSP90 (regulators of fusion of multivesicular bodies with the plasma membrane), and Flotillin-1 (lipid raft-associated protein for sorting in exosomes) were present in FB-eNVs, in addition to GM130 (a peripheral cytoplasmic protein that is tightly bound to Golgi membranes) and calnexin (an endoplasmic reticulum (ER) lectin), which were present in FB-eNVs as they were engineered from cells (Figure 2A). TEM results showed that FB-eNVs were intact and cup-like, round, and approximately 100 nm in diameter (Figure 2B). The presence of vascular endothelial growth factor (VEGF)-a was confirmed in FB-eNVs (Figure S1). These findings showed that fibroblast cells were successfully used to generate FB-eNVs.

### 3.2. Cellular Uptake of FB-eNVs and Proliferation of DP Cells by FB-eNVs

**Figure 3:**
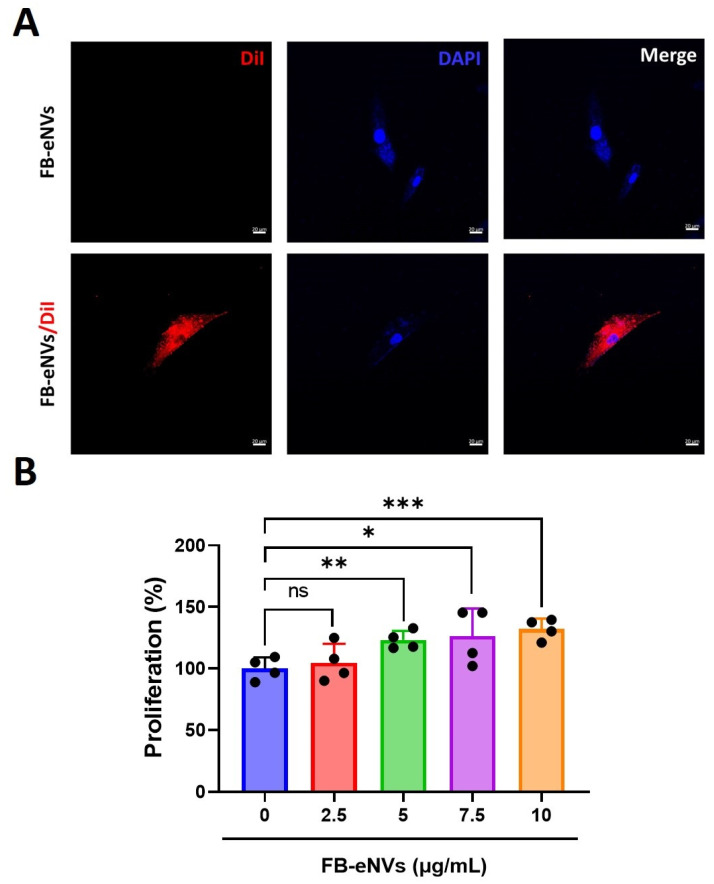
Cellular uptake of FB-eNVs into DP cells and proliferation of DP cells by FB-eNVs. (**A**) In vitro uptake of fluorescent stain (DiI)-labeled FB-eNVs to DP cells at 3 h. No FB-eNVs were detected in the control samples. The cell nucleus was stained with DAPI. Scale bar, 20 µm. (**B**) Percentage of proliferation of DP cells (*n* = 4) at 24 h after treatment with FB-eNVs (0 to 10 µg/mL). The values are mean ± sd of a minimum of three replicates of an experiment. Student’s *t*-test was used. * *p* < 0.05; ** *p* < 0.01; and *** *p* < 0.001. ns: not significant.

To examine the internalization of FB-eNVs, 10 µg of FB-eNVs was conjugated with Dil dye and incubated with DP cells. Fluorescence microscopy imaging demonstrated that FB-eNVs were actively internalized into DP cells (Figure 3A). The effects of FB-eNVs (2.5–15 µg/mL of FB-eNVs) on the proliferation of DP cells were examined, and the results showed that FB-eNV treatment significantly increased the proliferation of DP cells (*p* < 0.05) with 5 to 15 µg/mL of FB-eNVs (Figure 3B). This result indicated that the internalization of FB-eNVs induced the proliferation of DP cells.

### 3.3. FB-eNVs Promote the Migration of DP Cells

**Figure 4:**
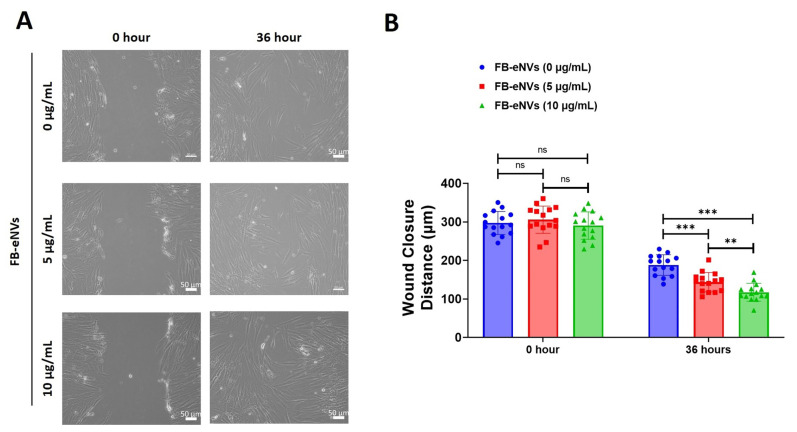
FB-eNVs promoted the migration of DP cells. (**A**) Phase-contrast microscopy images of migrated cells at 0 and 36 h after FB-eNV (5 and 10 µg/mL) treatment. Scale bar: 50 µm. (**B**) Percentage of wound closure (*n* = 16) at 0 and 36 h after FB-eNV (5 and 10 µg/mL) treatment. The values are mean ± sd of a minimum of three replicates of an experiment. Student’s *t*-test was used. ** *p* < 0.01; and *** *p* < 0.001. ns: not significant.

To determine the effects of FB-eNVs on DP cell migration, we used FB-eNVs. DP cells showed significantly increased migration at 5 µg/mL and 10 µg/mL FB-eNVs (*p* < 0.001) compared with the effect observed in the control samples. DP cells showed significantly increased migration at 10 µg/mL (*p* < 0.01) of FB-eNVs compared to 5 µg/mL FB-eNVs (Figure 4A,B), indicating that FB-eNVs can induce the migration of DP cells.

### 3.4. FB-eNV Treatment Modulates Signaling Pathways in DP Cells

**Figure 5:**
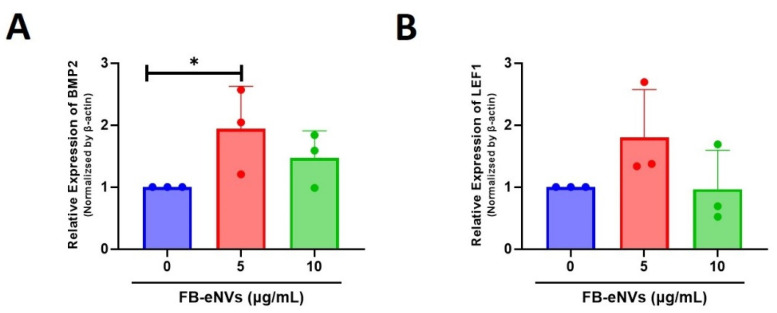
FB-eNV treatment modulated the mRNA expression in DP cells. (**A**,**B**) Relative mRNA expression of BMP2 and LEF1 in DP cells, 24 h after FB-eNV (5 and 10 µg/mL) treatment; β-actin was used as an internal control. The values are mean ± sd of a minimum of three replicates of an experiment. Student’s *t*-test was used. * *p* < 0.05.

**Figure 6:**
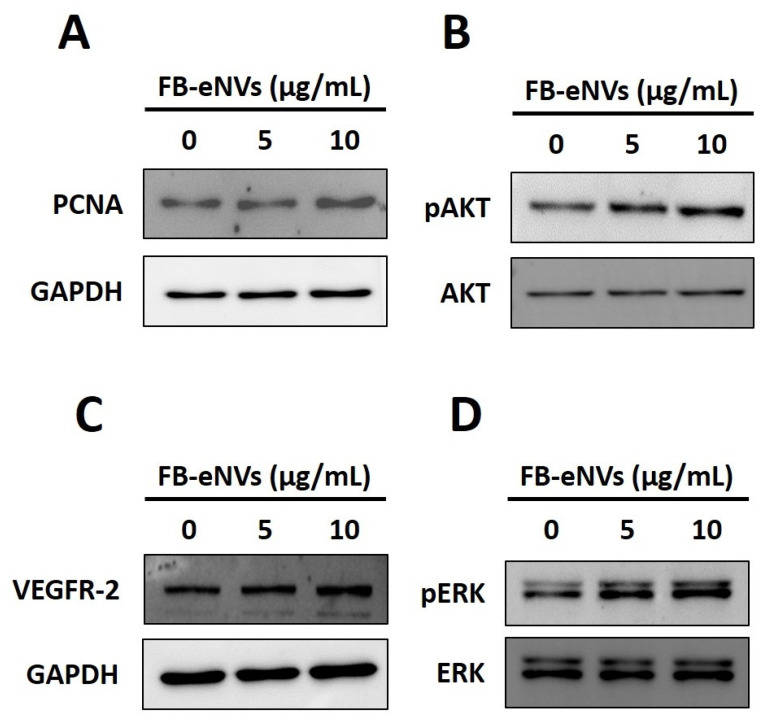
FB-eNV treatment modulated signaling pathways in DP cells. (**A**–**D**) Western blot analysis of PCNA, pAKT, AKT, VEGFR-2, pERK, ERK, and GAPDH levels in DP cells after 24 h of treatment with FB-eNVs.

To determine the effects of FB-eNVs on the activity of cellular signaling pathways in DP cells, we first examined the mRNA expression of BMP2 and LEF1. The expression of BMP2 was increased 1.94-fold (*p* < 0.05) and 1.47-fold with 5 µg/mL and 10 µg/mL FB-eNV treatments, respectively (Figure 5A). The expression of LEF1 was increased 1.80-fold with 5 µg/mL FB-eNV treatment, with no significant change in the 10 µg/mL FB-eNV treatment (Figure 5B). The expression of the proliferation marker (PCNA) was increased after treatment with FB-eNVs when compared with control samples (Figure 6A). In addition, FB-eNV treatment increased the levels of phosphorylated AKT (pAKT) compared with those in controls (Figure 6B). The VEGFR-2 protein levels increased in a dose-dependent manner compared with those in controls (Figure 6C). Furthermore, FB-eNV treatment increased the levels of phosphorylated ERK (pERK) (Figure 6D), indicating that FB-eNVs influence the signaling pathways in DP cells.

### 3.5. FB-eNV Treatment Increased Hair Shaft Elongation

**Figure 7:**
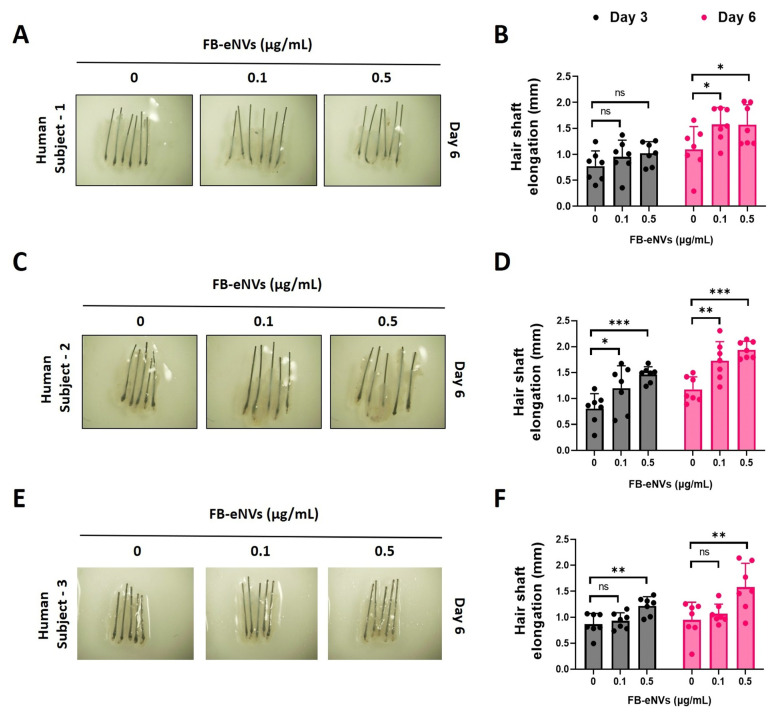
FB-eNV treatment promoted hair shaft elongation. (**A**,**C**,**E**) Representative images of human hair follicles after FB-eNV treatment (0, 0.1, and 0.5 µg/mL) at day 6. (**B**,**D**,**F**) Quantified data of hair shaft elongation on day 3 and day 6 (*n* = 6–7). The values are mean ± sd of a minimum of three replicates of an experiment. Student’s *t*-test was used. * *p* < 0.05; ** *p* < 0.01; and *** *p* < 0.001. ns: not significant.

To examine the elongation of hair shafts, mini-organ cultures were created using human scalp HFs. HFs were treated with FB-eNV (0, 0.1, and 0.5 μg/mL) for 6 days; the results showed that FB-eNVs only slightly increased the length of the hair shaft at day 3 and significantly (*p* < 0.05) compared with controls on day 6 in human subject 1 (Figure 7A,B). FB-eNVs significantly increased the hair shaft length (*p* < 0.05, *p* < 0.01, and *p* < 0.001) compared with controls on days 3 and 6 in human subject 2 (Figure 6C,D). Furthermore, FB-eNVs significantly increased the hair shaft length (*p* < 0.01) compared with controls on days 3 and 6 in human subject 3 (Figure 7E,F). These results revealed that FB-eNV increased the growth of hair shafts in human HFs.

## 4. Discussion

Advances in our understanding of the generation of hair fibers and their regulation by cyclic hair follicles have opened the door to the safe treatment and effective enhancement of hair growth. DP cells, which originate from mesenchymal components, conventionally release growth factors to induce and maintain the development of HFs, regulate the hair cycle, and regenerate HFs. Therefore, DP cells are considered the key signaling centers [7,17]. In this study, we successfully engineered NVs from fibroblasts and found evidence of the direct role of FB-eNVs in DP cells and HFs.

Fibroblasts were selected to generate eNVs as they are intricately associated with the development and maintenance of skin and HFs [18,19]. To generate FB-eNVs, fibroblasts were extruded through a serial-sized membrane using a mini-extruder, filtered, and purified. Studies have reported that EVs possess certain biomarkers based on their biogenesis [20]; however, eNV biomarkers have not been extensively explored. Therefore, we confirmed that three of the classical EV protein markers (Alix, HSP90, and Flotillin-1), often used to characterize EVs [20,21], were expressed in FB-eNVs. In addition, cellular proteins such as GM130 and calnexin are present in FB-eNVs as the FB-eNVs are synthesized through direct cell extrusion and may contain all organelle components [22], including the Golgi apparatus and ER. Furthermore, biological cargoes (including proteins) are randomly packed into eNVs, rather than the selective packing of specific proteins in EVs [23,24,25]. Nonetheless, EVs and eNVs (also called EV-mimetics) have been demonstrated to have 80% similar proteomes [22]. Next, we confirmed that the generated FB-eNVs were intact/round and approximately 100 nm in diameter, and within the range of typical EVs (e.g., exosomes) [3,26].

Most studies, including our previous studies, have reported that EVs/NVs exert their therapeutic effects on hair growth by activating the Wnt/β-catenin signaling pathways [7]. BMP2 and LEF1 are the known DP signature genes in mice and humans, respectively [27]. BMP2 is one of the members of the BMP family that is a powerful regulator of skin and hair development [28]. LEF-1 is known as a target gene for Wnt signaling as β-catenin alone cannot activate the expression of Wnt target genes without co-transcription factors, and LEF1 is one of the co-transcription factors [29]. Moreover, Wnt signaling is required to maintain the trichogeicity of DP cells and control hair cycling [27,30,31,32,33]. We evaluated the expression of known target genes in the BMP (BMP2) and Wnt (LEF1) pathways [27], and their expression was increased in DP cells following FB-eNV treatment. EVs deliver different biological materials to recipient cells to influence them; therefore, we checked VEGF-a in FB-eNVs because VEGF has been demonstrated to induce the proliferation of DP cells in HFs by activating VEGFR-2 [34,35]. Western blotting results showed that FB-eNVs carry material from VEGF-a in their compartments.

EVs can communicate with recipient cells by interacting with cell membrane receptors or by delivering material to cells [36,37]. EVs (exosomes, EVs, and eNVs) first interact with the plasma membrane, followed by their internalization in different ways, such as clatherin- or calveolin-mediated endocytosis, macropinocytosis, phagocytosis, and lipid raft-mediated and direct fusion, to permit the delivery of biological materials into recipient cells [38,39,40]. The proliferation of DP cells is required for the morphogenesis and growth of HFs [41,42], and FB-eNVs have been demonstrated to interact with DP cells and internalize into cells, which is in agreement with other studies [7]. The interaction and internalization of FB-eNVs induce the proliferation of recipient DP cells, which agrees with the findings of previous studies showing that EVs/NVs induce DP cell proliferation. Furthermore, we found that FB-eNVs increased the migration of DP cells. Previous studies have demonstrated that EVs derived from MSCs, macrophages, and DP cells increase the migration of DP cells [43,44,45].

In the present study, because FB-eNV treatment induced the proliferation of DP cells, we assessed the levels of PCNA in DP cells after FB-eNV treatment, and the results showed increased PCNA levels in DP cells. Furthermore, the levels of pAKT, which is known to induce the proliferation and migration of DP cells [7,43,44], increased in DP cells after FB-eNV treatment. VEGF can induce the proliferation of DP cells through the VEGFR-2-mediated activation of ERK [34,35,46]. Western blotting results showed that the treatment of DP cells with FB-eNVs increased the levels of VEGFR-2 and pERK, suggesting that the VEGF-a present in FB-eNVs could have enabled the VEGFR-2-mediated activation of pERK, thereby affecting the proliferation and migration of DP cells. Several studies have demonstrated that the survival rate of cells is determined by AKT/ERK, which plays a pivotal role in mediating survival signals [44,47,48,49]. Further studies are required to understand the nature of the molecules responsible for this function and their modes of action.

Because we could not assess the effect of FB-eNVs in humans, we used HFs isolated from patients with androgenic alopecia (three different individuals). We studied them under ex vivo conditions for their effect on hair shaft elongation, which closely mimics clinical situations and is commonly used in preclinical studies. Our ex vivo experimental results suggest that FB-eNVs elongated the hair shafts in all individuals.

## 5. Conclusions

The current study demonstrated that engineered NVs can be successfully isolated and purified from fibroblasts and can induce hair growth both in vitro and ex vivo. Thus, FB-eNVs could be a desirable therapeutic candidate for large-scale clinical production. Moreover, this can considerably reduce the cost of production.

## Data Availability

Not applicable.

## Supplementary Materials

The following supporting information can be downloaded at: https://www.mdpi.com/article/10.3390/cells11244066/s1, Figure S1. Presence of VEGF-a in FB-eNVs. Western blot analysis of fibroblasts and FB-eNV lysates for the detection of VEGF-a; Ponceau S staining served as a loading control.
