# Supplementary material for: Engineered Nanovesicles from Fibroblasts Modulate Dermal Papillae Cells In Vitro and Promote Human Hair Follicle Growth Ex Vivo
Source: Cells. 2022 Dec 15;11(24):4066. doi: 10.3390/cells11244066 (PMC9777471; doi:10.3390/cells11244066)
Supplement: Supplementary file 1 [file cells-11-04066-s001.zip › cells-1930964-supplementary.pdf]

cells

Article

Engineered Nanovesicles from Fibroblasts Modulates Dermal Papillae Cells In Vitro and Promote Human Hair Follicle Growth in Ex Vivo

Ramya Lakshmi Rajendran [1,†], Prakash Gangadaran [1,2,†], Mi Hee Kwack [2,3], Ji Min Oh [1], Chae Moon Hong [1,4], Madhan Jeyaraman [5,6], Young Kwan Sung [2,3], Jaetae Lee [1,4], Byeong-Cheol Ahn [1,2,4,*]

[1] Department of Nuclear Medicine, School of Medicine, Kyungpook National University, Daegu, Republic of Korea.
[2] BK21 FOUR KNU Convergence Educational Program of Biomedical Sciences for Creative Future Talents, Department of Biomedical Sciences, School of Medicine, Kyungpook National University, Daegu, Republic of Korea.
[3] Department of Immunology, School of Medicine, Kyungpook National University, Daegu, Republic of Korea.
[4] Department of Nuclear Medicine, Kyungpook National University Hospital, Daegu, Republic of Korea.
[5] Department of Orthopaedics, Faculty of Medicine - Sri Lalithambigai Medical College and Hospital, Dr MGR Educational and Research Institute University, Chennai, Tamil Nadu, India.
[6] Department of Biotechnology, School of Engineering and Technology, Sharda University, Greater Noida, Uttar Pradesh, India.
* Correspondence: abc2000@knu.ac.kr; Tel.: 82-53-420-5583
† These authors contributed equally to this work.

Supplementary Materials:

Citation: Lastname, F.; Lastname, F.; Lastname, F. Title. *Cells* **2022**, *11*, x. https://doi.org/10.3390/xxxxx

Academic Editor: Firstname Lastname

Received: date
Accepted: date
Published: date

Publisher's Note: MDPI stays neutral with regard to jurisdictional claims in published maps and institutional affiliations.

Copyright: © 2022 by the authors. Submitted for possible open access publication under the terms and conditions of the Creative Commons Attribution (CC BY) license (https://creativecommons.org/licenses/by/4.0/).

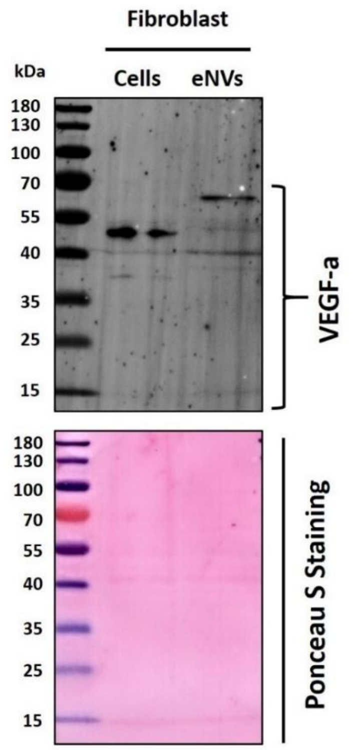

Figure S1. Presence of VEGF-a in FB-eNVs. Western blot analysis of fibroblasts and FB-eNV lysates for the detection of VEGF-a; Ponceau S staining served as a loading control.

Table Supplementary S1. PCR primers used in this study.

Gene	Oligonucleotide primers	
β-actin	GCACAGAGCCTCGCCTT	GTTGTCGACGACGAGCG
LEF1	CAGGAGCCCTACCACGACAA	CCTCCATCTGGATGCTTTCC
BMP2	Hs-BMP2-1-SG (QuantiTect Primer, Qiagen)	
